# Psychometric properties of the positive mental health questionnaire: short form (PMHQ-SF18) in young adults

**DOI:** 10.3389/fpubh.2024.1375378

**Published:** 2024-05-10

**Authors:** Carlos Sequeira, José Carlos Carvalho, Juan Roldan-Merino, Antonio R. Moreno-Poyato, Sónia Teixeira, Beatriz David, Patrício Soares Costa, Montserrat Puig-Llobet, Maria Teresa Lluch-Canut

**Affiliations:** ^1^Nursing School of Porto (ESEP), Porto, Portugal; ^2^Center for Research in Health Technologies and Services: Health Research Network, From The Lab to The Community (CINTESIS@RISE), ESEP Hub, Porto, Portugal; ^3^Campus Docent Sant Joan de Déu, Sant Boi de Llobregat, Spain; ^4^Study Group on Invariance of Instruments for Measurement and Analysis of Change in the Social and Health Environment, Barcelona, Spain; ^5^Departament d’Infermeria de Salut Pública, Salut Mental i Materno-Infantil, Escola d’Infermeria, Facultat de Medicina i Ciències de la Salut. Grup de Recerca de Cures Infermeres en Salut Mental, Psicosocials i de Complexitat (NURSEARCH – 2021 SGR 1083), Universitat de Barcelona, Barcelona, Spain; ^6^Higher School of Health Fernando Pessoa (ESS-FP), Porto, Portugal; ^7^Faculty of Psychology and Education Sciences, University of Porto, Porto, Portugal; ^8^School of Medicine, University of Minho, Braga, Portugal; ^9^Life and Health Sciences Research Institute (ICVS), School of Medicine, University of Minho, Braga, Portugal; ^10^ICVS/3B’s - PT Government Associate Laboratory, Braga, Portugal

**Keywords:** positive mental health, nursing, health promotion, factor analysis, statistical

## Abstract

**Introduction:**

Positive Mental Health (PMH) plays a pivotal role in the promoting of mental health. Assessing this phenomenon is essential for early recognition and intervention in mental health. To date, only one tool was validated with 39 items to assess PMH among Portuguese young adults.

**Method:**

This study sought to examine the psychometric properties of the short version of the Positive Mental Health Questionnaire (PMHQ) among Portuguese university students. The PMHQ Short Form was administered to a sample of 3,647 university students via an online platform. Exploratory and confirmatory factor analyses were performed. The principal factor solution was employed because some items showed higher levels of kurtosis. Multivariate analysis was tested using the Mardia’s Test, Henze-Zirkler, and Royston. Findings of content, construct validity tests, and Cronbach’s alfa demonstrated the satisfactory validity and suitable reliability of the PMHQ-Short Form (PMHQ-SF).

**Results and discussion:**

The exploratory factor analysis produced six dimensions of the PMHQ-SF with three items in each factor demonstrating adequate internal reliability. The global internal consistency was 0.92, with factors ranging between 0.60 to 0.82. The results suggest that the PMHQ-SF is reliable, easier, and more practical to complete by university students due to the shortening of the number of items. The PMHQ-SF is useful for assessing positive mental health in young adults. The final version of the instrument contains from 32 to 18 items.

## 1 Introduction

The term positive mental health, first designated by Marie Jahoda in her report, emphasizes the significance of promotion in modern societies and the dynamic life cycle of people (1). This perspective underscores the importance of maintaining health and fostering positive aspects of one’s life.

Positive mental health is conceptualized as the mental health of healthy people, achieved through the optimization of general well-being for optimal functioning of each human being across the life cycle, which is not a static state but a dynamic process (2). Furthermore, positive mental health is a compelling protective element against mental disorders (3).

Promotion brings health gains to societies, and evidence suggests that positive mental health acts as key resilience factor against suicide (4). Additionally, positive mental health serves as a protective factor that can reduce the risks of addictive excessive social media use (5) and is a well-known protective factor against psychological distress and anxiety symptoms (6).

Mental health problems are among the most significant causes of illness worldwide (7). Adolescence, in particular, is a critical period where mental health disorders become notably pronounced. Many studies indicate a rising prevalence of mental health problems among adolescents across various countries (8).

In Portugal, mental and behavioral disorders constitute 11.8% of the overall burden of disease, surpassing oncological diseases (10.4%) and trailing only behind cerebrovascular diseases (13.7%) (9). These statistics are alarming, highlighting the need to address the 89.2% of the Portuguese population without mental illness to strengthen their resistance and mental well-being (2).

In 2022, the prevalence of positive mental health among different university Portuguese students was 67.8% for a high level of PMH, 31.6% for a medium level of PMH, and 0.6% for a low level of PMH (10). Another study carried out in 2017, revealed that university students were mainly at the moderate level (67.7%) of PMH, 16.6% at the low level, and 15.7% at the high level (11).

These findings align with other recent studies demonstrating good levels of positive mental health (12–14) despite the current reality.

Consequently, the first step is to use validated psychometric instruments tailored to the population under study as a means of investing in prevention. Secondly, it is deemed important to simplify the available psychometric instruments facilitating their use.

The Positive Mental Health Questionnaire (PMHQ) is a self-administered questionnaire with 39 items, originally developed in Spanish (15) and validated for the Portuguese population (16). The items vas distributed into the six factors of the Multifactorial Model of Positive Mental Health. The six factors include *Personal Satisfaction* (F1) referring to satisfaction with oneself (self-concept/self-esteem), with personal life and future prospects; *Prosocial Attitude* (F2), including the person’s sensitivity to one’s social environment, the attitude and desire to support others, and the acceptance of others and differentiating social facts; *Self-control* (F3) including the person’s ability to deal with stress and conflict situations, emotional balance and tolerance to frustration, anxiety and stress; *Autonomy* (F4) comprising the person’s ability to make decisions by applying personal criteria, self-regulating of self-behavior and maintaining a good level of personal safety; *Problem Solving and Self-Actualisation* (F5) referring to the person’s ability to make decisions and solve the problems that life entails and the ability to adapt to changes, developing a flexible attitude and continuous personal growth; and *Interpersonal Relationship Skills* (F6) including the person’s ability to communicate and establish harmonious interpersonal relationships with the surrounding environment and the ability to communicate feelings and give and receive affection (15, 17, 18).

In the PMHQ, values between 39 (minimum value) and 156 (maximum value) can be obtained, and the higher the value obtained, the greater the global level of positive Mental Health. Thus, different global levels of Positive Mental Health can be categorized: low Level or *Languishing* for scores between 39 and 78, intermediate Level for scores between 79 and 117, and high Level or *Flourishing* for scores between 118 and 156. The PMHQ allows for obtaining global and factor scores. Respondents are asked to answer based on the frequency that best characterizes their case, choosing from options between “Always or almost always,” “Most of the time,” “Sometimes,” and “Rarely or never.” Of these 39 items, 19 are formulated positively, and 20 items are formulated negatively. The responses presented in a Likert-type scale will produce different scores or values (2).

Therefore, this study sought to develop and validate the Positive Mental Health Questionnaire Short-Form (PMHQ-SF), an instrument intended to assess positive mental health in adults based on the previously validated Positive Mental Health Questionnaire – PMHQ.

## 2 Methods

### 2.1 Participants

The sample comprised Portuguese university students from 19 different institutions across the country and different areas of training, with a predominance of nursing students (54.7%). Initially, 3,647 participants were involved, but 110 were excluded because due to having at least one missing response in the Positive Mental Health Questionnaire (PMHQ), and ab additional 15 were excluded for failing to provide gender information. The final sample of 3,522 participants was randomly split into two groups: a calibration sample and a validation sample.

### 2.2 Measures

#### 2.2.1 Positive mental health questionnaire

The scale was initially developed by Lluch-Canut with 39 items loaded into six factors: F1-Personal Satisfaction (items 4, 6, 7, 12, 14, 31, 38, 39), F2-Prossocial Attitude (items 1, 3, 23, 25, 37), F3-Self-Control (items 2, 5, 21, 22, 26), F4-Autonomy (items 10, 13, 19, 33, 34), F5-Problem Solving and Self-Realization (15, 16, 17, 27, 28, 29, 32, 35, 36), and F6-Interpersonnal Relationship Skills (items 8, 9, 11, 18, 20, 24, 30). The score ranges from 39 to 156. This scale shows good psychometric properties, with only Factor 2 presenting a lower Cronbach’s alpha (0.60) (15, 17).

The factorial structure of the scale was examined in a sample of Portuguese students (16), revealing good psychometric properties.

### 2.3 Analysis

Statistics analyses were conducted utilizing SPSS and R. One sample (*N* = 1,768) underwent exploratory factor analysis (EFA), and the other sample (*N* = 1,679) was subjected to confirmatory factor analysis (CFA). The selection criteria based on factor loadings in the EFA and CFA, with the highest chosen items. Oblimin rotation was applied for in EFA due to a correlation between factors suggested in previous studies. The factoring method selected was the principal factor solution, considering higher levels of kurtosis observed in some items. Multivariate analysis was conducted using three strategies: Mardia’s Test, Henze-Zirkler, and Royston.

An exploratory factor analysis (EFA) was conducted on the underlying structure of the measured positive mental health measured and subsequently, performed a confirmatory factor analysis (CFA). Measurement invariance was accessed by comparing models with increasing constraints to determine the degree of equivalence. In the configural model, the equivalence of structure without constraints was accessed; in metric invariance, loadings were constrained; in scalar invariance, the intercepts were compared; and for strict invariance, the model residuals were examined. A significant chi-square difference when comparing models indicated a lack of measurement invariance and a difference of CFI higher than-0.01 and RMSEA of 0.01 (19). The average variance extracted (AVE) was used to test convergent validity between constructs, with values above 0.70 considered very good and values below 0.50 indicating the difficulty in separating variance due to the construct itself and the one from measurement error (20).

## 3 Results

All variables exhibited acceptable skewness and kurtosis values, with item 3 presenting the highest kurtosis value in both the calibration and validation samples, namely 8.49 and 8.07, respectively, and skewness of -2.82 and -2.76, respectively (Table 1).

**Table 1:** Descriptive statistics for the validation and calibration sample.

	Calibration sample						Validation sample	Original sample
	*M*	*SD*	*Min*	*Max*	*Sk*	*K*	*M*	*SD*	*Min*	*Max*	*Sk*	*K*	*M*	*SD*	*Min*	*Max*	*Sk*	*K*
PMH 1	3.39	0.68	1	4	−0.96	0.84	3.39	0.70	1	4	−1.08	1.15	3.39	0.69	1	4	−1.03	1.03
PMH 2	3.01	0.79	1	4	−0.62	0.14	3.01	0.80	1	4	−0.67	0.24	3.02	0.8	1	4	−0.66	0.21
PMH 3	3.76	0.57	1	4	−2.82	8.49	3.74	0.60	1	4	−2.76	8.07	3.75	0.59	1	4	−2.80	8.39
PMH 4	2.94	0.86	1	4	−0.31	−0.76	2.91	0.87	1	4	−0.31	−0.76	3.93	0.87	1	4	0.31	−0.75
PMH 5	2.65	0.87	1	4	−0.07	−0.70	2.67	0.85	1	4	−0.08	−0.65	3.65	0.86	1	4	0.07	−0.67
PMH 6	3.11	0.79	1	4	−0.77	0.38	3.07	0.81	1	4	−0.76	0.34	3.09	0.8	1	4	−0.76	0.37
PMH 7	3.31	0.79	1	4	−1.04	0.59	3.25	0.84	1	4	−1.07	0.60	3.28	0.82	1	4	−1.06	0.65
PMH 8	3.51	0.72	1	4	−1.54	2.12	3.50	0.74	1	4	−1.53	2.02	3.51	0.73	1	4	−1.55	2.14
PMH 9	3.29	0.80	1	4	−1.03	0.63	3.29	0.79	1	4	−1.04	0.69	3.3	0.79	1	4	−1.04	0.67
PMH 10	2.99	0.91	1	4	−0.66	−0.33	3.01	0.91	1	4	−0.72	−0.22	3	0.91	1	4	−0.70	−0.27
PMH 11	3.07	0.83	1	4	−0.54	−0.38	3.03	0.84	1	4	−0.40	−0.73	4.05	0.84	1	4	0.47	−0.57
PMH 12	3.40	0.78	1	4	−1.32	1.36	3.37	0.81	1	4	−1.23	0.97	3.39	0.79	1	4	−1.28	1.17
PMH 13	3.17	0.75	1	4	−0.74	0.47	3.18	0.74	1	4	−0.78	0.62	3.18	0.75	1	4	−0.77	0.56
PMH 14	3.42	0.82	1	4	−1.37	1.17	3.43	0.80	1	4	−1.35	1.15	3.42	0.81	1	4	−1.37	1.19
PMH 15	3.32	0.75	1	4	−0.88	0.23	3.34	0.75	1	4	−0.92	0.31	4.33	0.75	1	4	0.90	0.28
PMH 16	2.81	0.93	1	4	−0.24	−0.90	2.78	0.94	1	4	−0.21	−0.93	3.79	0.93	1	4	0.22	−0.91
PMH 17	3.55	0.67	1	4	−1.33	1.18	3.53	0.67	1	4	−1.30	1.12	4.54	0.67	1	4	1.32	1.16
PMH 18	3.12	0.77	1	4	−0.56	−0.16	3.10	0.77	1	4	−0.46	−0.43	4.11	0.77	1	4	0.51	−0.30
PMH 19	2.94	0.89	1	4	−0.57	−0.36	2.95	0.88	1	4	−0.63	−0.23	2.94	0.89	1	4	−0.60	−0.30
PMH 20	2.84	0.93	1	4	−0.29	−0.87	2.85	0.91	1	4	−0.27	−0.86	3.85	0.92	1	4	0.28	−0.87
PMH 21	2.68	0.89	1	4	−0.03	−0.85	2.75	0.87	1	4	−0.08	−0.81	3.72	0.88	1	4	0.06	−0.82
PMH 22	2.77	0.82	1	4	−0.09	−0.68	2.81	0.80	1	4	−0.09	−0.68	3.79	0.81	1	4	0.09	−0.66
PMH 23	3.76	0.53	1	4	−2.28	5.20	3.74	0.54	1	4	−2.25	5.22	4.75	0.53	1	4	2.27	5.27
PMH 24	3.40	0.75	1	4	−1.25	1.37	3.40	0.74	1	4	−1.20	1.21	3.41	0.74	1	4	−1.24	1.33
PMH 25	3.28	0.74	1	4	−0.72	−0.13	3.26	0.75	1	4	−0.63	−0.40	4.27	0.74	1	4	0.68	−0.27
PMH 26	2.70	0.80	1	4	−0.13	−0.48	2.72	0.80	1	4	−0.14	−0.48	3.7	0.8	1	4	0.13	−0.48
PMH 27	3.27	0.70	1	4	−0.53	−0.45	3.26	0.71	1	4	−0.59	−0.22	4.27	0.7	1	4	0.57	−0.30
PMH 28	3.08	0.78	1	4	−0.42	−0.50	3.07	0.79	1	4	−0.40	−0.59	4.08	0.78	1	4	0.41	−0.55
PMH 29	2.71	0.79	1	4	0.03	−0.63	2.70	0.80	1	4	−0.04	−0.58	3.7	0.8	1	4	−0.01	−0.60
PMH 30	3.13	0.90	1	4	−0.80	−0.17	3.11	0.90	1	4	−0.77	−0.25	3.12	0.9	1	4	−0.79	−0.19
PMH 31	3.55	0.76	1	4	−1.83	2.93	3.53	0.78	1	4	−1.76	2.55	3.54	0.76	1	4	−1.80	2.78
PMH 32	3.38	0.72	1	4	−0.89	0.17	3.36	0.74	1	4	−0.90	0.17	4.37	0.73	1	4	0.90	0.19
PMH 33	3.57	0.69	1	4	−1.70	2.76	3.57	0.70	1	4	−1.74	2.73	3.58	0.7	1	4	−1.74	2.85
PMH 34	2.81	0.87	1	4	−0.57	−0.22	2.80	0.89	1	4	−0.57	−0.30	2.81	0.88	1	4	−0.57	−0.25
PMH 35	2.95	0.92	1	4	−0.40	−0.85	2.97	0.91	1	4	−0.40	−0.83	3.96	0.92	1	4	0.40	−0.84
PMH 36	3.32	0.71	1	4	−0.67	−0.28	3.35	0.70	1	4	−0.74	−0.11	4.34	0.7	1	4	0.71	−0.18
PMH 37	3.72	0.55	1	4	−2.07	4.44	3.68	0.58	1	4	−1.78	2.80	4.7	0.56	1	4	1.93	3.61
PMH 38	3.16	0.89	1	4	−0.92	0.11	3.15	0.88	1	4	−0.92	0.19	3.15	0.89	1	4	−0.92	0.15
PMH 39	2.96	0.92	1	4	−0.68	−0.30	2.92	0.96	1	4	−0.64	−0.49	2.94	0.94	1	4	−0.66	−0.40

Mardia’s test showed a violation of normality in both kurtosis and skewness (*p* < 0.001 for both). The chi-square test showed no differences between the calibration and validation in the distribution of gender and age. For this reason, principal axis factoring was applied for exploratory factor analysis and robust maximum likelihood was conducted for confirmatory analysis. The loadings of each item in the exploratory factor analysis are detailed in Table 2, with the items retained in the short form being highlighted.

**Table 2:** Factor loadings for the exploratory factor analysis.

PMHQ Items			Factor loading				
	1	2	3	4	5	6	h2
SMP31_	0.75	0.07	0.01	0.01	0.04	−0.02	0.59
SMP38_	0.72	−0.07	0.03	0.02	0.01	0.06	0.59
SMP14_	0.64	0.03	0.00	0.19	0.01	−0.02	0.58
SMP12_	0.62	0.07	0.04	0.03	0.05	0.04	0.50
SMP39_	0.58	−0.11	0.01	0.09	0.01	−0.02	0.40
SMP7_	0.55	0.12	−0.01	−0.04	0.01	0.23	0.48
SMP4_	0.49	−0.16	0.16	0.09	0.13	0.03	0.51
SMP8_	0.06	0.56	−0.01	0.03	0.09	0.22	0.48
SMP3_	0.06	0.56	0.00	0.05	0.08	−0.05	0.33
SMP24_	−0.03	0.44	0.02	0.12	0.13	0.03	0.26
SMP1_	0.02	0.27	0.10	0.19	−0.03	0.02	0.14
SMP21_	0.02	0.00	0.83	−0.06	−0.03	0.03	0.67
SMP22_	−0.06	0.02	0.75	0.05	0.08	0.00	0.60
SMP5_	0.01	0.02	0.74	0.05	−0.07	−0.06	0.54
SMP26_	0.06	−0.10	0.52	0.05	0.13	0.14	0.51
SMP6_	0.33	0.16	0.38	−0.07	−0.09	−0.11	0.28
SMP2_	0.17	0.03	0.29	0.26	−0.07	0.12	0.38
SMP16_	0.17	−0.10	0.26	0.02	0.23	0.12	0.34
SMP19_	0.05	0.03	0.01	0.70	−0.05	−0.04	0.52
SMP10_	0.07	0.00	0.04	0.70	−0.04	−0.04	0.55
SMP13_	0.02	0.06	−0.01	0.67	0.03	0.05	0.50
SMP34_	0.13	−0.02	0.09	0.38	−0.05	0.26	0.41
SMP15_	−0.04	−0.07	0.08	0.38	0.32	0.09	0.37
SMP35_	−0.01	−0.08	0.04	0.34	0.28	0.02	0.25
SMP33_	0.08	0.22	−0.06	0.31	0.12	0.08	0.24
SMP36_	0.09	−0.15	0.05	0.12	0.60	0.04	0.51
SMP32_	0.22	0.04	0.01	−0.08	0.59	0.02	0.46
SMP17_	0.13	0.08	0.00	−0.02	0.54	0.01	0.37
SMP37_	−0.01	0.26	−0.05	−0.05	0.54	0.01	0.39
SMP25_	−0.05	0.27	0.08	−0.13	0.49	0.00	0.37
SMP23_	−0.01	0.19	0.03	0.11	0.47	−0.08	0.30
SMP27_	0.08	−0.14	0.23	0.09	0.45	0.12	0.50
SMP28_	0.09	−0.15	0.07	0.03	0.42	0.26	0.41
SMP18_	−0.10	0.24	0.05	0.03	0.39	0.22	0.36
SMP11_	−0.19	0.25	0.14	−0.02	0.36	0.00	0.24
SMP20_	0.03	−0.03	0.09	0.01	0.07	0.65	0.53
SMP9_	0.17	0.29	0.00	0.05	−0.12	0.52	0.50
SMP30_	0.19	0.11	−0.04	0.10	−0.03	0.33	0.25
SMP29_	0.02	−0.19	0.13	0.06	0.30	0.31	0.37

Only three items were selected for the short form, guided by the criteria of having the highest loadings in both EFA and CFA.

When the results of these two methods diverged, a model with both solutions was computed to identify the best fit. For factors F1, F3, and F4, the three items with higher loadings converging between EFA and CFA were selected for the short scale. For factor F1, items 14, 31, and 38 were retained; for factor F3, items 5, 21, and 22; and for factor F4, items 10, 13, and 19. Regarding factors F5 and F6, the two methods (e.g., EFA and CFA) produced different results, with only two of the three highest loading items being the same. For factor F5, the best items presented by the EFA were, in descending order of their factor loadings, items 32, 36, and 17. The CFA also showed items 32 and 26 to be amongst the highest loading but presented question 27 as the highest loading. When included in a short-form model tested by a CFA, 17 produced a better overall fit and was preferred to 27. Regarding factor F6, items 20 and 9 were identified in two of the highest loadings in both the EFA and CFA but for the other item to be retained, EFA had item 30 and CFA item 18. Again, the two items were included in a CFA of the final model, and item 30 exhibited a better fit. For factor F2, the CFA had items 23, 25, and 37 with the three highest loadings whereas, in EFA, only two of the four items were from the original factor (items 1 and 3).

In alignment with existing literature, it was decided to retain the items suggested by the CFA. Using a sample of Spanish university students, Roldan-Merino (21) verified that items 23, 25, and 37 had the highest loading when subjected to a CFA.

After comparing the results of both EFA and CFA, the final short scale was refined to include 18 items, with three items attributed to each factor (Table 2). To access the psychometric properties of the short scale, a CFA was conducted demonstrating a good fit, *χ*^2^ (120) = 580, *p* < 0.001, *N* = 1761, CFI = 0.97, TLI = 0.93, SRMR = 0.048, RMSEA = 0.045, *p* = 0.94, 90% CI [0.041, 0.049]. Reliability was tested using Cronbach’s alpha and MacDonald’s Omega, yielding acceptable results for each factor (a value higher than 0.60 was considered acceptable). The most problematic factor (Factor 2) exhibited the lowest reliability (0.60). The AVE values (Table 3) indicated convergent validity only for factors 1, 3, and 4.

**Table 3:** Reliability.

	Alpha	Omega	AVE
F1	0.82	0.82	0.60
F2	0.60	0.62	0.36
F3	0.82	0.83	0.61
F4	0.78	0.80	0.58
F5	0.68	0.69	0.42
F6	0.66	0.67	0.41

The invariance analysis revealed metric invariance based on the chi-square criteria, with the CFI and RMSEA difference criteria supporting full invariance.

An additional analysis was conducted to evaluate the correlation between the composite factor scores in the full and proposed short form of the PMHQ-The results displayed very high correlations for all six factors and the total score of the scale, as presented in Table 4.

**Table 4:** PMHQ short form invariance.

Model	*χ*2	Df	CFI	Δχ2	ΔCFI	ΔRMSEA
Configural	773	240	0.95			
Metric	787	252	0.95	13.5	0.000	−0.001
Scalar	854	264	0.94	67.3*	−0.005	0.002
Residual	951	282	0.94	97.4*	−0.008	0.001

*p < 0.001.

Finally, cut-off points for the short form were examined by establishing three categories (e.g., low, average, and high total PMH scores) based on a standard deviation criterion of 1 SD. Specifically, 530 participants were identified with low positive mental health scores (*M* = 48.83), 2,445 participants exhibited average scores (*M* = 56.56), and 547 participants revealed high positive mental health scores (*M* = 64.29).

## 4 Discussion

This study aimed to create a short-form version of the PMHQ (Supplementary Appendix I). Overall, the psychometric properties of this short version yielded good results. The overall reliability of the PMHQ-SF18 is 0.92, with a factor ranging from 0.60 to 0.82. There are many instruments to measure well-being or mental health (22), but only fewer addressing a specific area of mental health - positive mental health.

Concerning the internal consistency of the PMHQ-SF18, these study findings for the global scale demonstrated good reliability (0.92). The Global Cronbach’s alpha value identified in this study is slightly higher compared to that reported by Almubaddel (23) in Saudi Arabia (0.86), by Hasan et al. (24), in Bangladesh (0.85), by Naghavi et al. (25), in Persia (0.90), and slightly lower than that found by Lukat et al. (26) in a German sample (0.93), and by Yilmaz and Eldeleklioğlu (27) in Turkey, where scales are based on other theoretical models.

Notably, factor F2 (Prosocial Attitude) exhibited reliability issues. However, this finding aligns with other studies, where this factor consistently displayed the lowest Cronbach’s alpha (Table 5). Thus, the short version demonstrates similar levels of reliability and validity when compared to the extended version.

**Table 5:** Correlation matrix for the original and short form’s factors.

	F1	F2	F3	F4	F5	F6	Total	F1_S	F2_S	F3_S	F4_S	F5_S	F6_S	Tot_S
F1	1													
F2	0.23	1												
F3	0.58	0.24	1											
F4	0.59	0.20	0.46	1										
F5	0.54	0.42	0.61	0.45	1									
F6	0.45	0.51	0.37	0.35	0.50	1.00								
Total	0.82	0.51	0.76	0.70	0.83	0.71	1.00							
F1_S	0.92	0.20	0.50	0.58	0.48	0.41	0.75	1.00						
F2_S	0.17	0.86	0.21	0.10	0.46	0.45	0.45	0.14	1.00					
F3_S	0.49	0.22	0.94	0.36	0.54	0.31	0.67	0.41	0.20	1.00				
F4_S	0.52	0.14	0.41	0.92	0.38	0.25	0.60	0.50	0.05	0.32	1.00			
F5_S	0.45	0.42	0.48	0.33	0.87	0.46	0.70	0.40	0.46	0.43	0.26	1.00		
F6_S	0.50	0.31	0.38	0.39	0.43	0.82	0.65	0.47	0.26	0.31	0.30	0.38	1.00	
Tot_S	0.79	0.48	0.76	0.71	0.77	0.66	0.96	0.76	0.46	0.70	0.64	0.71	0.69	1.00

Moreover, the study by Sequeira et al. (16) involving Portuguese university students observed the same distribution of items originally from factor F2, with items 1 and 3 loading in the second factor, but items 23, 25, and 37 loading in factor F5. These results underscore that, since the original development of this questionnaire (17), this factor has revealed poor psychometric properties, and several studies advocate further evaluation in future research (17, 21). However, the significance of this conceptual factor and the results obtained in a validity analysis support retaining this factor (Table 6).

**Table 6:** PMHQ alpha studies.

Factor		Alpha	
	Lluch-Canut (2003)	Roldán-Merino (2019)	Sequeira (2014)
1. Personal satisfaction	0.83	0.79	0.84
2. Prosocial attitude	0.58	0.54	0.51
3. Self-control	0.81	0.77	0.84
4. Autonomy	0.77	0.75	0.77
5. Problem-solving and self-realization	0.79	0.78	0.84
6. Interpersonal relationship skills	0.71	0.64	0.69

The PMHQ-SF18 is a fast-filling instrument for measuring global positive mental Health, comprising six factors: Personal Satisfaction (F1), Prosocial Attitude (F2), Self-control (F3), Autonomy (F4), Problem-Solving and Self-Actualization (F5), and Interpersonal Relationship Skills (F6). It consists of 18 questions on a 4-level Likert frequency scale with scores ranging from 1 to 4.

For this analysis, the negative items must be inverted. A global PMHQ-SF18 score can be obtained from each of the six factors (minimum 3–12 maximum). The values of the global PMHQ-SF ranged between a minimum of 18 and a maximum of 72.

In this study, the issues found in factor F2 likely stemmed from poor cultural appropriation of the perception of helping others. This self-awareness of the need to set personal goals in promoting activities such as volunteering and unpaid functions in the community is not exactly rooted in the education priorities of the Portuguese population, which can affect the results of factor F2. Therefore, further insight and analysis of this issue are deemed pertinent.

This tool seems to overcome perceived barriers and facilitating factors to evaluate positive mental health, thereby addressing the imperative to support the development of effective mental health strategies (28). This is particularly crucial in the assessment phase and can pose distinct challenges across cultures. Another challenge to overcome was the short time for the application of the questionnaire (29), potentially interfering in the credibility of the answers. Therefore, this shortened tool proves to be quicker to administer.

### 4.1 Strengths and limitations

This study has several strengths. First, the substantial sample size enhances the interpretability of the results. Also, the outcomes hold significant relevance as the PMHQ-SF18 facilitates the assessment of mental health, thereby assisting health professionals across different settings in promoting mental health. Notwithstanding these results, certain limitations should be acknowledged. While these study results may be generalizable to young adult university students sharing similar socio-demographic characteristics, they may not to extend to populations with less homogeneous characteristics. Although these findings may offer valuable insights into the characteristics of the general population, it is imperative to validate these assumptions through further research.

## 5 Conclusion

The PMHQ-SF18 showed good psychometric properties, with reliability and validity values similar to those of the original extended version of the PMHQ. For this reason, the PMHQ-SF18 an effective instrument for measuring positive mental health among university students. Although this study has successfully demonstrated the reliability and validity of the PMHQ-SF18, further research involving larger samples is needed to provide additional scientific evidence supporting this short version.

## 6 Relevance for clinical practice

These study findings have noteworthy implications to inform mental health professionals about the availability of a shortened version of the Positive Mental Health Questionnaire. This study encourages mental health nurses and other professionals to incorporate the assessment of positive mental health into their practice using validated psychometric instruments. This short version with 18 items enhances its suitability for clinical practice, making it more practical and easier to apply.

Moreover, the social relevance of this study lies in its contribution to the advancement of positive mental health assessment and promotion strategies, particularly among young university adults. By providing a reliable and practical tool for assessing positive mental health, this study holds the potential to inform interventions that enhance the well-being and resilience of young adults, ultimately contributing to healthier and more supportive communities. The validation of a shorter version of the Positive Mental Health Questionnaire (PMHQ) among Portuguese university students fosters mental health promotion by offering a reliable and practical tool for assessing positive mental health. This study enhances early detection and intervention strategies by providing a culturally sensitive instrument that is accessible and feasible for young adults.

## Data availability statement

The datasets presented in this article are not readily available because the data that served as the basis for the article is on an institutional basis from the Porto Superior School of Nursing. Data may be requested through a formal request as per data protection regulations. Requests to access the datasets should be directed to carlossequeira@esenf.pt.

## Ethics statement

This study was authorized by the Ethics Committee of the Porto Higher School of Nursing (2023_1945) and the participants provided written informed consent to participate in this study.

## Author contributions

CS: Conceptualization, Data curation, Formal analysis, Funding acquisition, Methodology, Project administration, Resources, Validation, Visualization, Writing – original draft, Writing – review & editing. JC: Conceptualization, Data curation, Formal analysis, Funding acquisition, Methodology, Project administration, Resources, Validation, Visualization, Writing – original draft, Writing – review & editing. JR-M: Conceptualization, Investigation, Methodology, Resources, Supervision, Validation, Visualization, Writing – original draft, Writing – review & editing. AM-P: Conceptualization, Investigation, Methodology, Resources, Supervision, Validation, Visualization, Writing – original draft, Writing – review & editing. ST: Conceptualization, Investigation, Methodology, Resources, Supervision, Validation, Visualization, Writing – original draft, Writing – review & editing. BD: Data curation, Formal analysis, Methodology, Software, Writing – review & editing. PC: Data curation, Formal analysis, Methodology, Software, Writing – review & editing. MP-L: Conceptualization, Investigation, Methodology, Resources, Supervision, Validation, Visualization, Writing – original draft, Writing – review & editing. ML-C: Conceptualization, Investigation, Methodology, Resources, Supervision, Validation, Visualization, Writing – original draft, Writing – review & editing.
