# Supplementary material for: Psychometric properties of the positive mental health questionnaire: short form (PMHQ-SF18) in young adults
Source: Front Public Health. 2024 May 10;12:1375378. doi: 10.3389/fpubh.2024.1375378 (PMC11122894; doi:10.3389/fpubh.2024.1375378)
Supplement: Supplementary file 1 [file Table_1.pdf]

Appendix I

POSITIVE MENTAL HEALTH QUESTIONNAIRE - SHORT FORM (PMHQ-SF18)

(Sequeira et al., 2023)*

This questionnaire contains a series of statements about how you think, feel and act, which is more or less common for each individual. Please read each statement carefully and then provide your answer according to the frequency that best characterises you by ticking the answer options:

1-Always or almost always 2-Most of the time 3-Sometimes 4-Rarely or never

Item	**Descriptive**	**1**	**2**	**3**	**4**
**1**	I consider myself a less important person than the other people around me				
**2**	I think I am a trustworthy person				
**3**	I can control myself when I experience negative emotions				
**4**	I am very concerned about what people think of me				
**5**	I try to improve myself as a person				
**6**	I have difficulties establishing satisfactory interpersonal relationships with some people				
**7**	I think I am worthless and useless				
**8**	I think about others' needs				
**9**	I can control myself when I have negative thoughts				
**10**	The opinions of others have a major influence on my decision-making				
**11**	I try to develop and enhance my good attitudes				
**12**	I consider myself to be a sociable person				
**13**	I am dissatisfied with myself				
**14**	I like helping others				
**15**	I can maintain good self-control in conflict situations that arise in my life				
**16**	I worry that people will criticise me				
**17**	When I have a problem, I try to find possible solutions				
**18**	I find it difficult to relate openly to my teachers/leaders				

*This instrument's use is pending authorization from the original author
