# Supplementary material for: Psychometric properties of the positive mental health questionnaire: short form (PMHQ-SF18) in young adults
Source: Front Public Health. 2024 May 10;12:1375378. doi: 10.3389/fpubh.2024.1375378 (PMC11122894; doi:10.3389/fpubh.2024.1375378)
Supplement: Supplementary file 2 [file Table_2.pdf]

Appendix II - Distribution of items in the two versions

FACTORS	PMHQ		PMHQ-SF18	
**Factor 1**	Item no. 14	-	**Item no. 1***	-
**PERSONAL SATISFACTION**	Item no. 31	-	**Item no. 7***	-
	Item no. 38	-	**Item no. 13***	-
**Factor 2**	Item no. 23	+	**Item no. 2**	+
**PROSOCIAL ATTITUDE**	Item no. 25	+	**Item no. 8**	+
	Item no. 37	+	**Item no. 14**	+
**Factor 3**	Item no. 5	+	**Item no. 3**	+
**SELF-CONTROL**	Item no. 21	+	**Item no. 9**	+
	Item no. 22	+	**Item no. 15**	+
**Factor 4**	Item no. 10	-	**Item no. 4***	-
**AUTONOMY**	Item no. 13	-	**Item no. 10***	-
	Item no. 19	-	**Item no. 16***	-
**Factor 5**	Item no. 17	+	**Item no. 5**	+
**PROBLEM-SOLVING AND**	Item no. 32	+	**Item no. 11**	+
**SELF-ACTUALIZATION**	Item no. 36	+	**Item no. 17**	+
**Factor 6**	Item no. 9	-	**Item no. 6***	-
**INTERPERSONAL**	Item no. 20	+	**Item no. 12**	+
**RELATIONSHIP SKILLS**	Item no. 30	-	**Item no. 18***	-

Note: * indicates negative items
